# Artificial neural network (ANN) velocity better identifies benign prostatic hyperplasia but not prostate cancer compared with PSA velocity

**DOI:** 10.1186/1471-2490-8-10

**Published:** 2008-09-02

**Authors:** Carsten Stephan, Nicola Büker, Henning Cammann, Hellmuth-Alexander Meyer, Michael Lein, Klaus Jung

**Affiliations:** 1 Department of Urology, Charité – Universitätsmedizin Berlin, Germany; 2 Institute of Medical Informatics, Charité – Universitätsmedizin Berlin, Germany; 3 Berlin Institute for Urologic Research, Germany

## Abstract

**Background:**

To validate an artificial neural network (ANN) based on the combination of PSA velocity (PSAV) with a %free PSA-based ANN to enhance the discrimination between prostate cancer (PCa) and benign prostate hyperplasia (BPH).

**Methods:**

The study comprised 199 patients with PCa (n = 49) or BPH (n = 150) with at least three PSA estimations and a minimum of three months intervals between the measurements. Patients were classified into three categories according to PSAV and ANN velocity (ANNV) calculated with the %free based ANN "ProstataClass". Group 1 includes the increasing PSA and ANN values, Group 2 the stable values, and Group 3 the decreasing values.

**Results:**

71% of PCa patients typically have an increasing PSAV. In comparison, the ANNV only shows this in 45% of all PCa patients. However, BPH patients benefit from ANNV since the stable values are significantly more (83% vs. 65%) and increasing values are less frequently (11% vs. 21%) if the ANNV is used instead of the PSAV.

**Conclusion:**

PSAV has only limited usefulness for the detection of PCa with only 71% increasing PSA values, while 29% of all PCa do not have the typical PSAV. The ANNV cannot improve the PCa detection rate but may save 11–17% of unnecessary prostate biopsies in known BPH patients.

## Background

Prostate specific antigen (PSA) is accepted as screening test for prostate cancer (PCa) detection but has its limitations especially in test specificity [1]. To improve PSA specificity, many methods have been introduced, e.g. measurements of molecular forms of PSA like free PSA [2,3], PSA in relation to prostate volume (PSA density, PSAD) [4], age related reference PSA values [5] or PSA changes over time which is known as PSA velocity (PSAV) [6]. To date only the use of percent free PSA (%fPSA) has been clinically accepted to improve specificity [7].

Recently the clinical usefulness of PSAV has debated intensively. Some authors argue for a lower cutoff for PSAV of 0.4 ng/mL per year instead of the former 0.75 ng/mL per year cutoff especially in younger men [8]. Others introduced age adjusted PSAV and PSA cutoffs for biopsy indication [9]. For younger patients (age 50–59) the PSAV cutoff should be lowered to 0.4 ng/mL per year to improve specificity [9]. Ito et al. [10] described a yearly threshold of 0.3 ng/mL as the optimal cutoff value of PSAV if the initial PSA level is 1–1.9 ng/mL and 0.75 ng/mL if the initial PSA is 2–4 ng/mL. Berger et al. [11] found significant differences in PSAV between PCa and patients with no evidence of malignancy.

In contrast, two recent studies did not prove the additional value of PSAV over PSA alone [12,13]. When considering the large biological variability of PSA of up to 20% [14] or differences in PSA values regarding the used assay [15] this may lead to misinterpretation. Different values are even more obvious when considering %fPSA [16-18].

An improved PCa detection rate was shown when using multivariate models like logistic regression [19] or artificial neural networks [20] which include %fPSA, PSA, and partially patient age, prostate volume and other clinical factors as input variables. However, until now the parameter PSAV has not been included in such a multivariate model.

The aim of this study was to combine both methods, the ANN and PSAV, and to validate the diagnostic usefulness of this new model with regard to the differentiation between PCa and benign prostate hyperplasia (BPH). For that purpose, we compared the diagnostic usefulness of the conventional PSAV and other parameters with the so-called ANN velocity (ANNV) that included the PSA and free PSA velocity data into an ANN model.

## Methods

From a cohort of 2959 patients visiting the Department of Urology (Charité Hospital Berlin) with total PSA (tPSA) and free PSA (fPSA) measurements from 1996–2006, a total of 199 patients were included. The selection criteria for this PSAV and ANNV study were at least three PSA and fPSA measurements with a minimum of three months interval between two measurements before treatment. All serum samples were drawn before any prostate manipulation (or at least 3–4 weeks after an earlier manipulation) and centrifuged within 2–3 hours after sampling. The samples were analyzed immediately or stored at -20°C for no longer than 48 hours before assay. The study was carried out in accordance with the standards of the local ethics board and the Helsinki Declaration of 1975 as revised in 1996.

All 199 patients (44–85 years) had a histological proven diagnosis of PCa (n = 49) or BPH (n = 150) based on examination of tissue samples obtained by transrectal ultrasound (TRUS)-guided sextant (until 1999) or octant prostate biopsies. Additionally, the status of digital rectal examination (DRE), age, and prostate volume (measured by TRUS) were also available.

Total and free PSA were measured with the IMMULITE PSA and Free PSA kits (Diagnostic Products, Los Angeles, CA, USA). The analytical performance and comparisons to other PSA tests have been described earlier [18,21]. Prostate volume was determined by TRUS using the prolate ellipse formula. A DRE finding non-suspicious for cancer was defined as negative and a finding suspicious for cancer as positive. All patients had a complete data set on tPSA, %fPSA, age, prostate volume, and DRE status at the time of the last PSA and fPSA measurement. In 14 of the 150 BPH patients (9.3%) the ANNV was not calculated using the first but the first available complete data set (fPSA and tPSA at 2^nd ^or 3^rd ^measurement) since partially the fPSA was not measured when tPSA was less than 2 ng/mL (1996–1999) or less than 1 ng/mL (1999–2006).

PSA values were included in the velocity calculation using the formula: (last PSA – first PSA)/time interval in ng/mL/year). Based on this formula, a one year short-term-PSAV, which describes the PSAV within the last 12 months before diagnosis, was also calculated.

The ANNV was calculated analogous with the ANN output values instead of the PSA values by using the same formula (last ANN output – first ANN output/time interval). The ANN was constructed with the SPSS-module Neural connection 2.0 (SPSS) as described earlier [22]. The back-propagation network consists of one input layer with the five neurons tPSA, %fPSA, patient age, prostate volume, and DRE status. Each ANN contains one hidden layer with three neurons. Each ANN finally contains one output neuron representing the output value as the probability of PCa. The activation function for the hidden neurons was the tanh while the activation function for the output neuron was linear in the range 0 to 1 to get a value for the probability of PCa. Training of the ANN took place in 4 steps with 100 sweeps each of them. Stopping criteria were a RMS error less than 0.001 or a rate of 95% correct classified samples. The initial weights were set randomly to values between -1 and +1. Before training all variables were normalized to mean value 0 and standard deviation 1 and ordered randomly. To avoid over-fitting we used 10-fold cross-validation. During training always 10% of the data were used for internal validation.

The respective PSA- and ANN-follow ups were divided into three groups. Group 1 consisted of increasing values (PSA >0.75 ng/mL/year; ANN >4/year), group 2 of stable values (PSA -0.75 to 0.75 ng/mL/year; ANN -4 to 4/year), and group 3 of the decreasing follow up values (PSA <-0.75 ng/mL/year; ANN <-4/year). When analyzing the follow up of %fPSA only, it has been shown that due to the large variability between the measurements there is no usefulness at all for the parameter %fPSA velocity (data not shown).

Statistical calculations were performed with SPSS 14.0 for Windows (SPSS, Chicago, USA). We used the non-parametric Mann-Whitney U test and the Kruskal-Wallis test. The diagnostic validity of all parameters was evaluated by Receiver-operating characteristic (ROC) curve analysis. The areas under the ROC curves (AUCs) and the specificities at 90% and 95% sensitivity were compared by a nonparametric method using the software GraphROC 2.1 for Windows. Significance was defined as *P *< 0.05.

## Results

The median tPSA for the PCa patients at the time of diagnosis (last tPSA value) was 8.3 ng/mL (range 3.2–107 ng/mL). The BPH patients had a significantly lower (*P *< 0.0001) median tPSA value of 5.3 ng/mL (0.4–37.1 ng/mL). Descriptive data for all analyzed tPSA ranges 0–4, 4.1–10, 10.1–20 and >20 ng/mL for all PCa and BPH including the median tPSA, %fPSA and prostate volume are shown in Table 1. The median age for all patients was 68 years and the age distribution revealed no differences between the 4 tPSA groups for the PCa where the median age was 66 years (45–80 years). The median age for the BPH patients was somewhat but not significantly higher (68 years, range: 44–85, *P *= 0.1) but did also not differ between the 4 groups.

**Table 1:** Distribution of patients within the different PSA ranges and median values for tPSA, %fPSA and prostate volume for all patients

	All patients				PCa				BPH			
	
tPSA range (ng/mL)	number	tPSA (ng/mL)	%fPSA (%)	volume (ml)	number	tPSA (ng/mL)	%fPSA (%)	volume (ml)	number	tPSA (ng/mL)	%fPSA (%)	volume (ml)
0–4	47	2.1	19.4	40	2	3.5	17.2	36.5	45	2.1	19.5	40
4.1–10	94	5.6	15.9	44.5	24	5.8	14.3*	33.5*	70	5.6	17.4	50
10.1–20	50	13.6	11	49.1	20	13.5	9.7*	44.5*	30	13.9	13.7	59
>20	8	25.7	9	35.5	3	29.3	6.8*	35*	5	25.6	11	80
all	199	6.2	15.1	45	49	8.3*	10.9*	35*	150	5.3	16.7	47

*significantly different from BPH patients with *P *< 0.0001

Regarding the follow up of the PCa patients, the number of PSA and fPSA measurements ranged from 3 to 13 (median: 4.5) whereas the BPH patients had on average more PSA and fPSA measurements (range 3 to 22, median: 7). The distribution of the follow up related to the years before diagnosis of PCa or total follow up time for the BPH patients is shown in Table 2. The median follow up time for all patients was 3.4 years while PCa patients had a shorter median follow up (1.8 years) compared with BPH patients (4.2 years).

**Table 2:** PSA follow up for all patients for all patient groups

	all patients		PCa		BPH	
	
Follow up (years)	number	percentage in %	number	percentage in %	number	percentage in %
0.5 to 1	12	6	6	12	6	4
1 to 2	47	24	21	43	26	18
2 to 4	56	28	15	31	41	27
4 to 6	41	20.5	5	10	36	24
6 to 9	43	21.5	2	4	41	27

all	199	100	49	100	150	100

**Figure 1:**
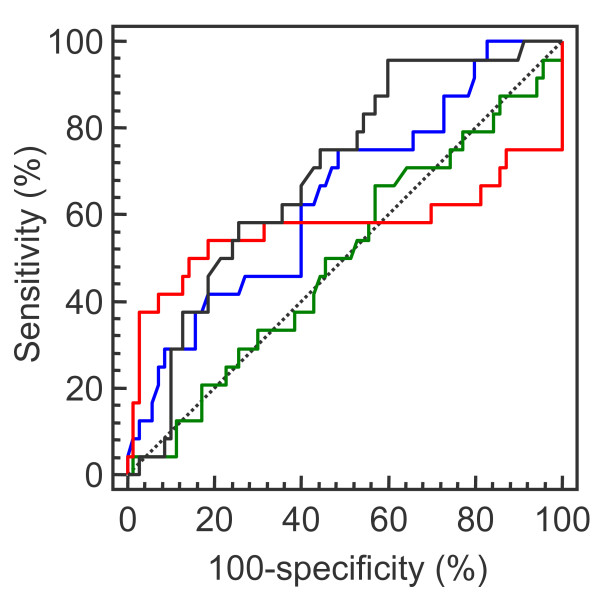
ROC curves for tPSA (green, AUC 0.5), %fPSA (blue, AUC 0.64), PSAD (black, AUC 0.69) and ANNV (red, AUC 0.57) to show the different behavior of the curve regardless of the AUC at tPSA 4–10 ng/mL (PSAV and ANN not shown, given in table 4).

Table 3 shows the ROC analysis for all 199 patients by comparing the AUC for tPSA, %fPSA, PSAD, PSAV, ANN output and the ANNV. PSAD was the best parameter to differentiate between PCa and BPH and neither ANN nor ANNV could improve this. At 95% sensitivity, PSAD performed better than all other parameters. On the other hand, at 95% specificity, the ANNV was the best available parameter with a sensitivity of 32.7% and significantly better performance compared with all others except %fPSA (*P *= 0.44). A similar behavior is seen for the 4–10 ng/mL tPSA range in Table 4. Again, regarding the AUC comparison and the specificities at 95% sensitivity, PSAD performed best, but did only reach significance levels to all others at 95% sensitivity but not for the AUC comparison. At 95% specificity, the ANNV (sensitivity 37.5%) demonstrated also within the tPSA range 4–10 ng/mL the ability to perform significantly better than all other parameters except the ANN output (*P *= 0.07). Figure 1 shows for the tPSA range 4–10 ng/mL that the ANNV has the steepest increase of the ROC curve with the highest sensitivities at 95% and 90% specificity, respectively. This may be more important for repeat biopsies, where biopsies in general should be avoided.

**Table 3:** Areas under the curves (AUC), specificities at 95% sensitivity and sensitivities at 95% specificity with the respective confidence intervals (in parenthesis) for the parameters tPSA, %fPSA, PSAD^§^, PSAV, ANN and ANNV^$ ^for all patients (n = 199)

Parameter	AUC (Confidence Intervals)	P-values and significance levels^§^	Specificity at 95% Sensitivity	P-values and significance levels^§^	Sensitivity at 95% Specificity	P-values and significance levels^$^
PSA	0.69 (0.61–0.77)	0.0001**	27.3 (21.4–34)	<0.0001***	14.3 (7–25.4)	0.008**
%fPSA	0.70 (0.71–0.78)	0.007**	17.3 (12.5–23.3)	<0.0001***	20.4 (11.6–32.3)	0.44
PSAD	0.76 (0.69–0.83)	-	44 (37.1–51.7)	-	16.3 (8.5–27.7)	0.013*
PSAV	0.76 (0.67–0.84)	0.835	4.7 (2.2–8.7)	<0.0001***	16.3 (8.5–27.7)	0.023*
ANN	0.66 (0.57–0.75)	0.001**	10 (6.3–15.1)	<0.0001***	18.4 (10–30)	0.023*
ANNV	0.56 (0.44–0.68)	<0.0001***	1.3 (0.2–4.3)	<0.0001***	32.7 (21.7–45.4)	-

^§^PSAD with largest AUC and highest specificity at 95% sensitivity, all others compared to PSAD

^$^ANNV with highest sensitivity at 95% specificity, all others compared to ANNV

*P < 0.05

**P < 0.01

***P < 0.0001

**Table 4:** Areas under the curves (AUC), specificities at 95% sensitivity and sensitivities at 95% specificity with the respective confidence intervals (in parenthesis) for the parameters tPSA, %fPSA, PSAD^§^, PSAV, ANN and ANNV^$ ^for the tPSA range 4–10 ng/mL (n = 94)

Parameter	AUC (Confidence Intervals)	P-values and significance levels^§^	Specificity at 95% Sensitivity	P-values and significance levels^§^	Sensitivity at 95% Specificity	P-values and significance levels^$^
PSA	0.50 (0.36–0.63)	<0.0001***	11.4 (5.9–19.8)	0.002**	4.2 (1.3–19.1)	0.037*
%fPSA	0.64 (0.51–0.77)	0.09	20 (12.6–29.6)	0.045*	12.5 (3.5–29.7)	0.023*
PSAD	0.69 (0.58–0.81)	-	35.7 (26.2–46.2)	-	4.2 (1.3–19.1)	0.013*
PSAV	0.66 (0.53–0.80)	0.529	4.3 (1.2–11.0)	<0.0001***	12.5 (3.5–29.7)	0.023*
ANN	0.66 (0.53–0.79)	0.264	8.6 (3.8–16.4)	<0.0001***	16.7 (5.9–34.6)	0.074
ANNV	0.57 (0.40–0.75)	0.008**	0	<0.0001***	37.5 (21.2–56.4)	-

^§^PSAD with largest AUC and highest specificity at 95% sensitivity, all others compared to PSAD

^$^ANNV with highest sensitivity at 95% specificity, all others compared to ANNV

*P < 0.05

**P < 0.01

***P < 0.0001

In Table 5 the respective three groups for PSAV and ANNV (increasing, stable and decreasing values) are given. More than two third of all PCa patients have the typical increasing PSAV. In comparison, the ANNV is only indicated at 45% of all PCa patients' increasing values. The differences between the PSAV and ANNV are also given in the Table 5. It can be seen that only BPH benefit from the additional ANNV since the stable values are significantly more (+17.4%). Also, there is a reduction of increasing values (-10.6%). This avoids repeated prostate biopsies in at least 11% of all BPH patients. Another observation is that more than half of all patients (52%) show an atypical PSAV with regard to their diagnosis.

**Table 5:** Comparison of the PSA and ANN velocity in PCa and BPH patients

	PSA velocity (PSAV)		ANN velocity (ANNV)			
follow up group	PCa in % (n = 49)	BPH in % (n = 150)	PCa in % (n = 49)	Difference to PSAV	BPH in % (n = 150)	Difference to PSAV

increasing	71.4%	21.3%	44.9%	- 26.5%	10.7%	- 10.6%
stable*	18.4%	65.3%	34.7%	+ 16.3%	82.7%	+ 17.4%
decreasing	10.2%	13.3%	20.4%	+ 10.2%	6.7%	- 6.6%

*by using the cutoff of 0.75 ng/mL/year for tPSA (range -0.75 to 0.75) and by using the cutoff of 4 per year for ANN output (range -4 to 4)

When using the traditional PSAV cutoff of 0.75 ng/mL/year the regular median PSAV for the PCa patients was 1.24 ng/mL/year whereas the PSAV for the BPH patients was 0.16 ng/mL/year (*P *< 0.0001). Further descriptive data are given for the patients with increasing, stable and decreasing PSAV (Table 6) and ANNV (Table 7).

**Table 6:** Median values and p-values between the 3 groups of increasing, stable or decreasing PSAV values

	Increasing PSAV (>0.75 ng/mL/year)			Stable PSAV (-0.75 to 0.75 ng/mL/year)			Decreasing PSAV (< -0.75 ng/mL/year)		
Parameter	PCa (n = 35)	BPH (n = 32)	p-value	PCa (n = 9)	BPH (n = 98)	p-value	PCa (n = 5)	BPH (n = 20)	p-value

Age (years)	65	68	0.15	68	68	0.86	72	68.5	0.92
tPSA (ng/mL)	11.7*	11.5^*$^	0.72	4.8^§^	4.96	0.96	6.2*	4.39	0.067
%fPSA (%)	9.6^*§^	14.1	0.001	15	17.4	0.49	21	16	0.13
Volume (mL)	35	46.5	0.066	33	45	0.1	55	57.5	0.89

The p-value is given for the comparison between the respective PCa and BPH patients (Mann-Whitney U Test)

*significantly different to the respective patients in the stable group (*P *< 0.05; Mann-Whitney U Test)

^§^significantly different to the respective patients in the decreasing group (*P *< 0.05; Mann-Whitney U Test)

The Kruskal-Wallis Test for the PCa patients between all 3 groups showed for tPSA (p = 0.0003) and %fPSA (p = 0.0008) significant differences but not for age (p = 0.26) or volume (p = 0.17).

The Kruskal-Wallis Test for the BPH patients between all 3 groups showed for tPSA (p < 0.0001) significant differences but not for %fPSA (p = 0.4), age (p = 0.96) or volume (p = 0.44).

**Table 7:** Median values and p-values between the 3 groups of increasing, stable or decreasing ANNV values

	Increasing ANNV (> 4)			Stable ANNV (-4 to 4)			Decreasing ANNV (< -4)		
Parameter	PCa (n = 22)	BPH (n = 16)	p-value	PCa (n = 17)	BPH (n = 124)	p-value	PCa (n = 10)	BPH (n = 10)	p-value

Age (years)	65*	68.5	0.61	70	68	0.7	62	64	0.29
tPSA (ng/mL)	8.3	6.8	0.17	10.2	5.3	0.002	5.5	4.35	0.5
%fPSA (%)	8.0^*$^	13.15	0.017	15.15	17.4	0.85	11.2	13.8	0.1
Volume (mL)	33.5*	36.5*	0.34	50	53.5^$^	0.87	30.5*	34*	0.26

The p-value is given for the comparison between the respective PCa and BPH patients (Mann-Whitney U Test)

*significantly different to the respective patients in the stable group (*P *< 0.05; Mann-Whitney U Test)

^§^significantly different to the respective patients in the decreasing group (*P *< 0.05; Mann-Whitney U Test)

The Kruskal-Wallis Test for the PCa patients between all 3 groups showed only for %fPSA (p = 0.014) significant differences but not for age (p = 0.32), tPSA (p = 0.16) or volume (p = 0.98).

The Kruskal-Wallis Test for the BPH patients between all 3 groups showed for volume (p = 0.022) significant differences but not for age (p = 0.31), tPSA (p = 0.61) or %fPSA (p = 0.17).

When analyzing the short-term-PSAV over 12 months, the median PSAV for the PCa patients is higher with 1.61 ng/mL/year but the median PSAV for the BPH patients is almost zero with 0.04 ng/mL/year (*P *= 0.0001). There are only slight differences between PCa patients when looking at the PSAV for a 12 months period (data not shown). However, around one third of all BPH patients change the status of stable values which were visible over a long time observation to increasing or decreasing values when only calculating the PSAV over 12 months. Instead of 65% by using the regular PSAV, only 32% of all BPH patients had stable values when using the short-term-PSAV.

## Discussion

The discussion regarding the use of PSAV to improve the low specificity of PSA has gained increasing attention. Initially, Carter et al. [6] presented at a PSAV cut off of 0.75 ng/mL/year a specificity of 90% – significantly higher than the 60% specificity of a single PSA cutoff of 4 ng/mL. The results, though, were based on analyzing only 18 cancer cases [6]. In a current analysis on a large cohort of patients the authors found that the PSAV cutoff of 0.75 ng/mL/year underestimated the risk of PCa especially in younger men and recommended age-adjusted cutoffs [9]. However, this analysis excluded 45% of men (5,381 from 11,861 men) with a PSAV of 0 or less and 30% (504 from 1654) PCa patients without increasing PSAV. The remaining 70% PCa patients had an increasing PSAV, which is the same percentage of PCa patients as we found in our study with increasing PSA values (Table 5). However, in our analysis, all patients regardless of the PSAV were considered. On the other hand, 30% of all PCa patients do not present with the typical increasing PSA values. 10% of all PCa patients even have decreasing PSA values. This shows that PSAV can detect approximately 2/3 of all PCa patients but stable or decreasing PSA values do not really reduce the risk of having PCa if the PSA alone is elevated.

It was assumed that additional clinical and laboratory data would improve the PCa detection rate when using ANN models where %fPSA, age, prostate volume and the DRE status are considered. However, this study could not demonstrate a positive effect by using the ANNV to detect more cancer patients since only 45% of all PCa patient had an increasing ANNV. This is a reduction of 26.5% compared with the PSAV. The number of stable or decreasing ANNV output values increased compared with the PSAV indicating that PSAV alone is the better indicator for a PCa risk. More than half of all PCa patients had a stable or even decreasing ANNV. It should be noted that this poor performance of the ANN is only related to the follow up but not to the ANN use at all for PCa detection. Here it has been demonstrated that ANN models with clinical and laboratory values can significantly improve the PCa detection rate compared with PSA and %fPSA [20,22-25]. However, the relatively small number of patients with only one third PCa is a limitation of this study compared with other ANN studies where no follow up was analyzed.

Importantly, the inclusion of the ANNV can substantially save repeated biopsies in BPH patients. Whereas the PSAV shows only for 65.3% of the BPH patients the typical continuous follow up, this number increases to 82.7% when using the ANNV. Thus, at the best case approximately 17% of all BPH patients may benefit if taking into account not only the PSA but also the ANN follow up. When only looking at the difference between an increasing PSAV and increasing ANNV, the ANNV could save approximately 11% of all biopsies compared with the PSAV. As seen in Figure 1, the ANNV has the highest sensitivities at 95% and 90% specificity, respectively. Thus, relatively good sensitivity values at high specificity cutoffs argue for a usability of the ANNV especially for repeat biopsies, where biopsies in general should be avoided. This is an important result of the study. A further possibility is to look at partial ROC areas, which has been published before [26]. When only including the AUC between 80% and 100% specificity, the ANNV has clearly the largest AUC compared with all others. Hence, for a ROC comparison one should not only consider the AUC but also the ROC curve shape for a better interpretation.

Another problem is the relatively poor performance of the PSAV alone, which can be partially explained by the biological variation of PSA of up to 20% [14]. Differences in PSA values regarding the used assays may be also responsible [15]. However, this could be excluded in our study since only the IMMULITE assays were used for the tPSA and fPSA measurements over the whole time period from 1996 until 2006. The use of %fPSA revealed large differences between commonly used assays [16-18]. In a recent study on 4,480 men in 5 different populations with 5 different PSA and fPSA assays and the application of different assay-adapted ANNs it has been demonstrated that our recently multicentric evaluated ANN "ProstataClass" [22] should not be used without consideration of the PSA assay [25]. In another study, Okamura and colleagues [27] reported an acceptable comparability between two PSA assays by using a %fPSA-based logistic regression model.

To calculate the PSAV we subdivided the PSA follow ups into three categories with increasing (> 0.75 ng/mL/year), stable (-0.75 to 0.75 ng/mL/year) and decreasing (< -0.75 ng/mL/year) values. The same procedure was performed with the ANNV, where 4/year was taken as cutoff. Contrary to others [28], we found it difficult to further subdivide also the category of inconsistent values. We did not find it useful to determine a definitive cutoff for the ANNV as Carter et al. [6] did for the PSAV but the cutoff 4/year for the ANNV was taken for this preliminary study which equals to 90% specificity to have the possibility to discriminate between increasing, stable and decreasing values. However, the number of patients is relatively small and the usefulness of a cutoff especially for the PCa detection has not been shown.

Recently, a study has reported that different methods to calculate the PSAV either with simple arithmetic or linear regression does not change the outcome [29]. Data from this study were also not different when using the linear regression calculated compared with the arithmetic method (not shown).

Whereas the advantage of ANN models compared with PSA or %fPSA has been proven in many studies [20,22], the situation for PSAV compared with PSA is unclear [8,11-13,30,31]. Studies on large populations have shown a clear advantage for PSAV compared with PSA alone [8,11,31]. Apart from PSAV, the age and the tPSA range should be also considered [8,31]. Another study in screened men proved a significant difference for the PSAV between PCa (median: 0.62) and men with a negative biopsy (median: 0.46) but could not confirm a clinical advantage [30]. Furthermore, the studies by Thompson et al. [13] and Schroeder et al. [12] also on large populations, did not show any advantage of using PSAV instead of PSA. In a recent review on studies of PSAV it was explained why the association between PSAV and disease-specific survival, which has been shown in other studies, does not necessarily imply that PSAV will be a useful screening tool [32]. Moreover, other results show that patients who have a PSA which returns to normal levels still have a significant risk of PCa which led the authors to the conclusion that prostate biopsy might be most appropriate even after a single abnormal PSA [28]. Here we only partially agree because a simple repeated measurement of the PSA can avoid a significant number of biopsies and inclusion of ANN models give further certainty for a correct biopsy indication [33].

## Conclusion

To conclude, this study demonstrates limited usefulness of PSAV to detect PCa with only 71% of increasing PSA values while approximately 30% of all PCa do not have the typical PSA follow up. The ANNV cannot improve the PCa detection rate but may save 11–17% of unnecessary prostate biopsies in BPH patients. Further studies on screening and larger populations are needed to determine the usefulness of the ANNV.

## Abbreviations

ANN: artificial neural network; ANNV: ANN velocity; BPH: benign prostatic hyperplasia; DRE: digital rectal examination; fPSA: free PSA; PCa: prostate cancer; PSA: prostate-specific antigen; PSAD: PSA density; PSAV: PSA velocity; percent free PSA; tPSA: total PSA; TRUS: transrectal ultrasound

## Competing interests

The authors declare that they have no competing interests.

## Authors' contributions

All authors read and approved the final manuscript.

## Pre-publication history

The pre-publication history for this paper can be accessed here:


